# High-Efficiency Polarization Multiplexing Metalenses

**DOI:** 10.3390/nano12091500

**Published:** 2022-04-28

**Authors:** Xueping Sun, Rui Ma, Xinxin Pu, Shaobo Ge, Jin Cheng, Xiangyang Li, Quan Wang, Shun Zhou, Weiguo Liu

**Affiliations:** 1 Shanxi Province Key Laboratory of Thin Films Technology and Optical Test, School of Optoelectronic Engineering, Xi’an Technological University, Xi’an 710032, China; sunxueping@xatu.edu.cn (X.S.); puxinxin@st.xatu.edu.cn (X.P.); geshaobo@xatu.edu.cn (S.G.); chengjin36@xatu.edu.cn (J.C.); 2 School of Microelectronics, Xidian University, Xi’an 710071, China; marui@xatu.edu.cn; 3 National Key Laboratory of Science and Technology on Space Microwave, China Academy of Space Technology (Xi’an), Xi’an 710100, China; lixiangyang@sina.com; 4 Department of Biomedical Engineering, University of Strathclyde, Glasgow G1 1XQ, Scotland, UK; quan.wang.100@strath.ac.uk

**Keywords:** polarization multiplexing metalens, focusing efficiency, polarization, FWHM

## Abstract

The polarization multiplexing technique is a well-established method that improves the communication capacity of an optical system. In this paper, we designed orthogonal linear and circular polarization multiplexing metalens using a library of rectangle TiO_2_ nanostructures. The former can independently focus x- and y-linearly polarized incident lights to designed positions with a focusing efficiency of 53.81% and 51.56%, respectively, whereas the latter with two preset focal points can independently control left and right circularly polarized incident lights with a focusing efficiency of 42.45% and 42.46%, respectively. We also show that both metalenses can produce diffraction-limited focal spots for four polarization states with no obvious distortion, which opens up new applications in polarization imaging and polarization detection.

## 1. Introduction

In an imaging optical system, the optical elements can tailor the properties of light by imposing continuous phase retardation via their geometric and material properties [1]. To achieve high-quality images, it is necessary to utilize a complex assembly of optical elements that work together to mitigate aberrations and achieve an aesthetically pleasing image. The necessity of using these elements leads to a large-size and heavy-weight optical system, which limits their utility in size and weight-constrained applications. Recently, metasurfaces, which are composed of subwavelength-spaced nanostructures aligned on a planar substrate, have emerged as one of the leading platforms for the development of miniaturized optical components [2,3,4,5]. They can accurately adjust the amplitude, phase, and polarization state of the incident light through precise design of the geometrical parameters and the arrangements of each unit of nanostructures in a metasurface [6,7,8,9,10,11,12,13,14]. The advantages of flexible design, a light weight, flatness, and multifunction, make metasurfaces a viable substitute for the traditional bulky optical elements. To date, metasurfaces have been successfully applied in many fields, such as beam shaping [15], holography [9,16], and metalenses [17,18,19,20,21]. Among them, metalenses have gained considerable attention due to their planar, ultrathin, and miniaturized features. In addition, metalenses also show some unparalleled advantages compared to conventional lenses. For example, achromatic metalenses [22,23,24,25] working in a relatively broad wavelength bandwidth can be designed and achieved in many fields. Non-orthogonal polarization multiplexed metasurfaces have been proposed for tri-channel gray imaging [26] and polychromatic image displays [27].

In recent years, there has been a surge of interest in a novel metalens with polarization manipulation functionality. This metalens commonly utilized the propagation and geometric (or Pecharatnam-Berry, PB) phase to control the phase of left-circularly polarized (LCP) and right-circularly polarized (RCP) light. For instance, Wang et al. [28] designed a bifunctional metalens to focus the LCP and RCP light to different positions at a wavelength of 1500 nm. Then, they designed and achieved a metalens with four focal points through multiplexing two bifunctional metalenses [29]. Zhang et al. [4] designed a metalens with a tunable focal length by controlling the polarization state of the incident light, which possesses different longitudinal focal points for LCP and RCP light. Zhao et al. [30] designed two kinds of metalenses for orthogonal linearly and circularly polarized light based on amorphous silicon and fused quartz at a wavelength of 800 nm. Meanwhile, an orthogonal linear polarization multiplexing metalens, as another kind of metalens, has also been developed [6,31,32]. For example, a polarization-multiplexed metalens with a large numerical aperture was designed, which can independently control x- and y-polarized light [31]. Even though the two kinds of orthogonal polarization multiplexing metalenses have been achieved by many researchers, it is still a great challenge to design polarization multiplexing metalenses with good performance.

In this work, we report two kinds of polarization multiplexing metalenses that are constructed by a TiO_2_ nanostructures array based on a combining of the manipulations of propagation and geometric phases simultaneously We realized polarization multiplexing by engineering the phase of the orthogonal linear or circular polarizations simultaneously but independently at each position of the metasurface. Furthermore, we validated this by performing three-dimensional (3D) finite-difference time-domain (FDTD) simulations on two orthogonal polarization multiplexing metalenses. Both of the two metalenses exhibit good performance in focusing efficiency and full width at half maximum (FWHM) of the focal spots. This study marks a solid step toward the practical application of an integrated and miniaturized polarization imaging system.

## 2. Design and Method

The Jones vector is the efficient to describe the polarization state of a plane wave. For an arbitrary completely polarized light, it can be described as a superposition of any two orthogonal polarizations. There are two typical polarization expansion bases commonly used to describe this completely polarized light. One is a linear polarization expansion basis (|X〉,|Y〉), where |X〉 is the x linearly polarized vector and can be described in the form of the Jones vector as $\left[\begin{array}{c} 1\\ 0 \end{array}\right]$, where |Y〉 is the y linearly polarized vector and can be described in the form of the Jones vector as $\left[\begin{array}{c} 0\\ 1 \end{array}\right]$. Another one is the circular polarization expansion basis (|L〉,|R〉), where |L〉 is LCP and |R〉 is RCP. |L〉 and |R〉 can be expressed in terms of the linear polarization basis:$|L\rangle =\frac{1}{\sqrt{2}}\left(|X\rangle +i|Y\rangle \right)$,$|R\rangle =\frac{1}{\sqrt{2}}\left(|X\rangle -i|Y\rangle \right)$, where i is the imaginary unit.

**Figure 1:**
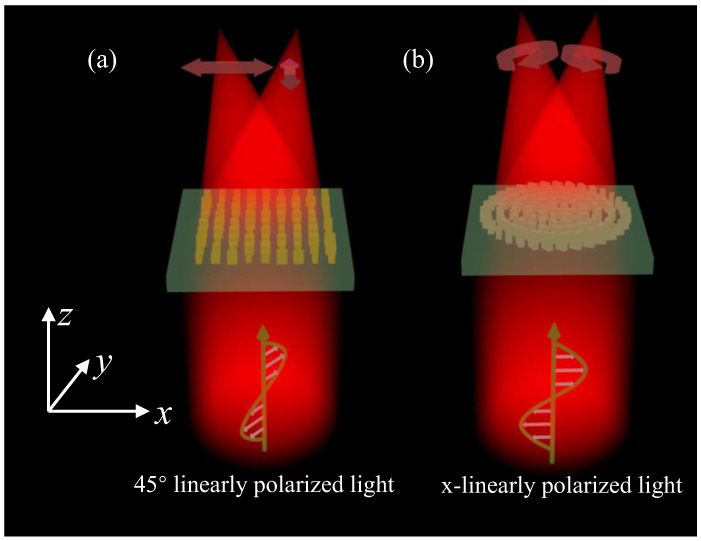
Schematic of the (**a**) orthogonal linear polarization multiplexing metalens and (**b**) orthogonal circular polarization multiplexing metalens.

This paper designed an orthogonal linear polarization multiplexing metalens and an orthogonal circular polarization multiplexing metalens based on the above two polarization expansion bases. Figure 1a shows the schematic of an orthogonal linear polarization multiplexing metalens that can focus x and y linearly polarized light to two spatially distinct focal spots under the illumination of a 45° linearly polarized light. The orthogonal circular polarization multiplexing metalens, which is shown in Figure 1b, can independently control RCP and LCP incident light. When an x-polarized light beam is delivered to the metalens, two focal spots for LCP and RCP will appear at their preset positions.

This focusing characteristic of the metalens mainly depends on the phase distribution of the unit nanostructure at each position. To design the orthogonal linear polarization multiplexing metalens with the focal spots at ($x_{1}$,$y_{1}$) and ($x_{2}$,$y_{2}$), the required phase profiles for x- and y-linearly polarized light can be, respectively expressed, respectively, as:

(1a) $$\varphi_{x}=\frac{2\pi }{\lambda }\left(\sqrt{f_{1}^{2}+x_{1}^{2}+y_{1}^{2}}-\sqrt{{\left(x-x_{1}\right)}^{2}+{\left(y-y_{1}\right)}^{2}+f_{1}^{2}}\right)$$

(1b) $$\varphi_{y}=\frac{2\pi }{\lambda }\left(\sqrt{f_{2}^{2}+x_{2}^{2}+y_{2}^{2}}-\sqrt{{\left(x-x_{2}\right)}^{2}+{\left(y-y_{2}\right)}^{2}+f_{2}^{2}}\right)$$

where x and y are the coordinate positions of each unit nanostructure, λ is the operation wavelength; $f_{1}$ and $f_{2}$ are the focal lengths for x- and y-linearly polarized light, respectively.

Correspondingly, the modulated phase profiles for the LCP and RCP incident light related to the orthogonal circular polarization multiplexing metalens should satisfy the following equations, respectively:

(2a) $$\varphi_{L}=\frac{2\pi }{\lambda }\left(\sqrt{f_{L}^{2}+x_{oL}^{2}+y_{oL}^{2}}-\sqrt{{\left(x-x_{0L}\right)}^{2}+{\left(y-y_{0L}\right)}^{2}+f_{L}^{2}}\right)$$

(2b) $$\varphi_{R}=\frac{2\pi }{\lambda }\left(\sqrt{f_{R}^{2}+x_{oR}^{2}+y_{oR}^{2}}-\sqrt{{\left(x-x_{0R}\right)}^{2}+{\left(y-y_{0R}\right)}^{2}+f_{R}^{2}}\right)$$

where ($x_{0L}$,$y_{0R}$) and $f_{L}$ are the offset and focal wavelength for LCP incident light, ($x_{0R}$,$y_{0R}$) and $f_{R}$ are the offset and focal length for RCP incident light.

**Figure 2:**
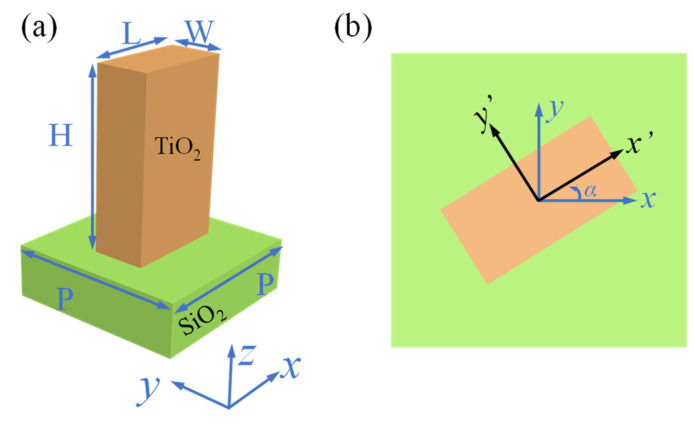
(**a**) A unit cell with a rectangular TiO_2_ nanostructure resting on SiO_2_. (**b**) A rotated unit cell.

The unit cells of the metalenses designed in this work are dielectric birefringent rectangle nanostructures that are embedded on a dielectric substrate. To acquire a high focusing efficiency, the materials of a unit cell should have negligible absorption at the designed wavelength. Here, we choose TiO_2_ and SiO_2_ as the materials of nanostructure and substrate, respectively. Figure 2a schematically depicts a unit cell with principal axes (length and width vectors) and x and y directions, respectively. When the length L and width W are different, a unit cell responds differently to phase shift and transmittance of x- and y-linearly polarized light.

Due to symmetry, a normally incident linearly polarized light along one of the principal axes does not change its polarization state and the phase and transmission coefficient can be acquired as it passes through this unit cell array [8]. This operation of a unit cell can be expressed using a Jones matrix as $J_{1}=\left[\begin{array}{cc} t_{x} & 0\\ 0 & t_{y} \end{array}\right]=\left[\begin{array}{cc} \exp\left(i\phi_{x}\right) & 0\\ 0 & \exp\left(i\phi_{y}\right) \end{array}\right]$. Here, we assumed unity transmission since the unit cells are highly transmissive. Figure 2b shows the rotated unit cell, whose Jones matrix can be written as [33]:

(3) $$J=R\left(-\alpha \right)J_{1}R\left(\alpha \right)$$

Here, $R\left(\alpha \right)=\left[\begin{array}{cc} \cos\left(\alpha \right) & \sin\left(\alpha \right)\\ -\sin\left(\alpha \right) & \cos\left(\alpha \right) \end{array}\right]$ is the rotation matrix by an angle α in a counterclockwise direction.

When α=0, the Jones matrix of a unit cell can be recombined as:

(4) $$J=\exp\left(i\phi_{x}\right)\left[\begin{array}{cc} 1 & 0\\ 0 & 0 \end{array}\right]+\exp\left(i\phi_{y}\right)\left[\begin{array}{cc} 0 & 0\\ 0 & 1 \end{array}\right]$$

The matrices $\left[\begin{array}{cc} 1 & 0\\ 0 & 0 \end{array}\right]$ and $\left[\begin{array}{cc} 0 & 0\\ 0 & 1 \end{array}\right]$ are responding to the Jones matrices of an ideal linear polarizer oriented with its transmission axis parallel to the *x* axis and *y* axis, respectively. The phase delay $\phi_{x}$ of a unit cell that responded to x-linearly polarized light is sensitive to L, and the phase delay $\phi_{y}$ of a unit cell that responded to y-linearly polarized light is sensitive to W. In designing an orthogonal linear polarization multiplexed metalens, we adopted the propagation phase modulation method, in which the propagation phase can be regulated by changing the unit cell length L and width W. With the correct choice of the TiO_2_ nanostructure height and the period, the unit cells can provide full and independent 2π phase control over x- and y-linearly polarized light, where x and y are aligned the principal axes of the unit cell. Furthermore, it allowed for the design of a metalens that controls x- and y-linearly polarized light imaging independently.

We also can represent the Jones matrix of the unit cell as:

(5) $$J=\exp\left(i\frac{\phi_{x}+\phi_{y}}{2}\right)\left\{\cos\left(\frac{\phi_{x}-\phi_{y}}{2}\right)\left[\begin{array}{cc} 1 & 0\\ 0 & 1 \end{array}\right]+\frac{i}{2}\sin\left(\frac{\phi_{x}-\phi_{y}}{2}\right)\exp\left(2i\alpha \right)\left[\begin{array}{cc} 1 & i\\ i & -1 \end{array}\right]+\;\frac{i}{2}\sin\left(\frac{\phi_{x}-\phi_{y}}{2}\right)\exp\left(-2i\alpha \right)\left[\begin{array}{cc} 1 & -i\\ -i & -1 \end{array}\right]\right\}$$

which can be divided into three terms:

(1) The first term is a unit matrix, which cannot change the polarization state of an incident light.
(2) The second term is $\frac{1}{2}\left[\begin{array}{cc} 1 & i\\ i & -1 \end{array}\right]$; both of its two eigenvectors are $\frac{1}{\sqrt{2}}\left[\begin{array}{c} 1\\ i \end{array}\right]$ (responding to |L〉), but the eigenvalue responding to the eigenvector is zero. In other words, the polarization state for the emerging beam will convert to its orthogonal polarization state $|R\rangle =\frac{1}{\sqrt{2}}\left[\begin{array}{c} 1\\ -i \end{array}\right]$. The conversion efficiency of this unit cell is not only dependent on $\frac{\phi_{x}-\phi_{y}}{2}$ but also on the polarization state of the incident light. And the conversion efficiency reaches its maximum value of 100% when $|\phi_{x}-\phi_{y}|=\pi$ and the polarization state of the incident light is |R〉. Here, a unit cell with $|\phi_{x}-\phi_{y}|=\pi$ can be functionalized as a half-wave plate [34].
(3) The third matrix is similar to the second and it can change the polarization state of an incident light to LCP. The conversion efficiency is 100% when $|\phi_{x}-\phi_{y}|=\pi$ and the polarization state of the incident light is LCP.

When passing through a unit cell with $|\phi_{x}-\phi_{y}|=\pi$, the incident LCP and RCP transformed into their orthogonal polarization state and obtained the same propagation phase $\phi_{p}=\left(\phi_{x}+\phi_{y}\right)/2$ and an opposite geometric phase [29] 2α. The propagation phase and PB phase can be controlled independently by changing the geometry and the rotation angle of a unit cell, respectively. Thus, the independent phases of LCP and RCP incident light can be achieved through the linear combination of the propagation and PB phase. Meanwhile, in the design of the orthogonal circular polarization multiplexing metalens, the propagation phase $\phi_{p}$ and PB phase 2α are employed to realize the phase profiles $\varphi_{L}$ and $\varphi_{R}$. Considering that $\varphi_{L}=\phi_{p}+2\alpha$ and $\varphi_{R}=\phi_{p}-2\alpha$, the required propagation phase and PB phase can be expressed as $\phi_{p}=\left(\varphi_{L}+\varphi_{R}\right)/2$, $\alpha =\left(\varphi_{L}-\varphi_{R}\right)/4$. Therefore, a series of unit cells with propagation phases ranging from 0 to 2π is required to achieve a complete phase coverage for $\phi_{p}$.

## 3. Simulation and Discussion

In this work, both of the two kinds of orthogonal polarization multiplexing metalenses with the same library of unit cells are operated at a 632.8 nm wavelength. We simulated and optimized the unit cells using the finite difference time domain (FDTD) method. If the phase change of $\phi_{x}$ and $\phi_{y}$ can range from 0 to 2π, a 2π phase coverage of $\phi_{p}$ also can be satisfied automatically. To ensure high efficiency, the period (P) and height (H) of a unit cell should be optimized at the wavelength we used. Based on empirical and trial methods, we set the period (P) 400 nm and height (H) 800 nm. The period (P) meets the Nyquist sampling criterion [18] $P<\frac{\lambda }{2NA}$, where NA is the numerical aperture of a metalens. The height of 800 nm is enough to meet 2π phase coverage for $\phi_{x}$ and $\phi_{y}$.

**Figure 3:**
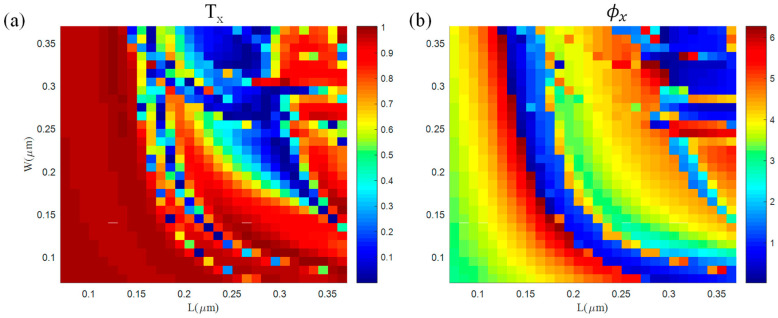
(**a**) Transmittance and (**b**) phase delay for x-linearly polarized light incidence with different lengths L and widths W.

Additionally, the length (L) and width (W) vary from 70 to 370 nm at intervals of 10 nm to obtain the transmittance and phase of the transmitted electric field. Here, we established a model of a unit cell with periodic boundary conditions in the x- and y-directions and perfectly matched layer (PML) boundary conditions in the z-direction. Figure 3a,b show the transmittance Tx and the phase $\phi_{x}$ obtained from the propagation of x-linearly polarized light passing through the unit cell. We can obtain the transmittance Ty and the phase $\phi_{y}$ by transposing the distribution matrix of Tx and $\phi_{x}$, respectively. From Figure 3b, it can be seen that the $\phi_{x}$ range covers 0~2π. Therefore, it satisfies the requirement of phase modulation. The phase $\phi_{x}$ and Tx are presented in Tables S1 and S2 in the Supplementary Materials.

**Figure 4:**
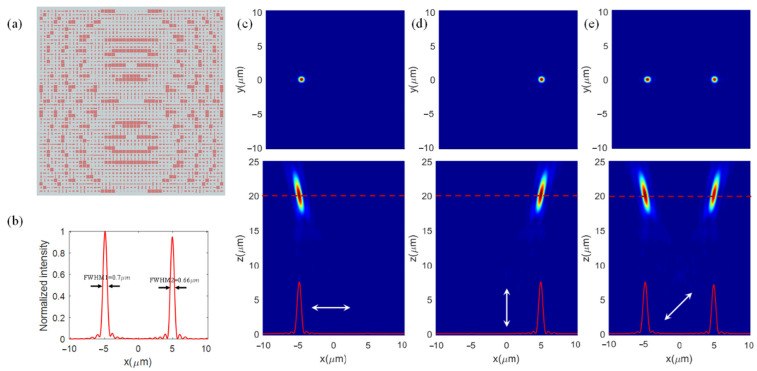
Simulation for the orthogonal linear polarization multiplexing metalens. (**a**) Top view of this designed metalens. (**b**) The sectional intensity profile along the *x*-axis in the focal plane. Intensity maps in the xy-plane and xz-plane under (**c**) x-, (**d**) y- and (**e**) 45°-linearly polarized light incidences.

Based on the discussion above, we have designed a linear orthogonal polarization multiplexing metalens. We set the focal length of this metalens as f1 = 20 um, f2 = 20 um, x1 = −5 um, x2 = 5 um, y1 = 0, and y2 = 0. According to Equation (1a,b), the phase modulation distribution for x- and y-linearly polarized light can be obtained. Figure 4a shows the top view of the designed metalenses, which consists of 51 × 51 unit cells with different lengths and widths. In the simulation model of the metalens, we used the total field scattered field (TFSF) source as the light source, which is different from the simulation model of a unit cell using a plane wave as its light source. All the boundary conditions are PML along the x-, y-, and z-directions. We selected the unit cells with a transmittance higher than 85% to form a database. The best unit cells with good performance at their position (*x_i_*,*y_i_*) can be optimized in this database by minimizing $\left|\phi_{x}\left(x_{i},y_{i}\right)-\phi_{x}^{C}\right|+\left|\phi_{y}\left(x_{i},y_{i}\right)-\phi_{y}^{C}\right|$. $\phi_{x}\left(x_{i},y_{i}\right)$ and $\phi_{y}\left(x_{i},y_{i}\right)$ are the required phases at (*x_i_*,*y_i_*) determined by Equation (1a,b). $\phi_{x}^{C}$ and $\phi_{y}^{C}$ are the phase delays of the optimal unit cell for x- and y-linearly polarized incident light.

To evaluate the focusing performance, the transmitted fields of the metalenses are calculated numerically. For the x-linearly polarized light incidence, the transmitted conserved x-linearly polarized light was focused at a position of (−5 um, 0 um, 20 um), shown in Figure 4c. We defined the focusing efficiency as the ratio of power around the focal spot within the circle having a radius of 3 times the FWHM to the power of the light source.

As shown in Figure 4b, the FWHM of the focal spot is 700 nm, which is close to the diffraction-limited 707 nm (0.5λ/NA). The focusing efficiency is 53.81% for the x-linearly polarized light incidence. It is below our expectations because the transmittance on the focal plane is only 69.82%. Therefore, we defined another parameter, diffraction efficiency, as the ratio of power around the focal spot within the circle having a radius 3 times the full width at half maximum (FWHM) to the total power on the focal plane. The diffraction efficiency reaches 77.08% for the x-linearly polarized light. For the y-linearly polarized light incidence, the transmitted conserved y-polarized light was focused at a position of (5 um, 0 um, 20 um), illustrated in Figure 4d. The FWHM is 660 nm and the focusing efficiency and diffraction efficiency are 51.56% and 75.48%, respectively. The 45°-linearly polarized light can be decomposed into x- and y-linearly polarized light with the same amplitude. Therefore, there are two focal spots at the preset positions for the 45°-linearly polarized light incidence, as shown in Figure 4e.

**Figure 5:**
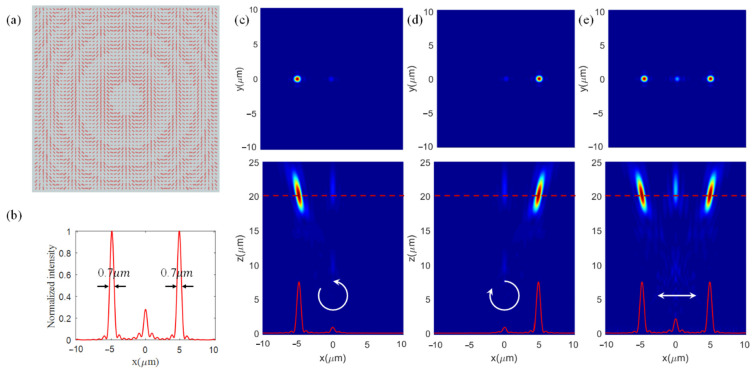
Simulation for the orthogonal circular polarization multiplexing metalens. (**a**) Top view of this designed metalens. (**b**) The sectional intensity profile along the *x*-axis in the focal plane. Intensity maps in the xy-plane and xz-plane under (**c**) LCP, (**d**) RCP, and (**e**) x-linearly polarized light incidences.

As for the circular orthogonal polarization multiplexing metalens we designed, it has the following parameters, *f_L_* = 20 um, *f_R_* = 20 um, *x_oL_* = −5 um, *x_oR_* = 5 um, *y_oL_* = 0, and *y_oR_* = 0. Similar to the linear orthogonal polarization multiplexing metalens, the phase modulation distribution for LCP and RCP incidence light can be calculated. However, this metalens has an issue with polarization conversion, which has been discussed in Section 2. We also select 51 × 51 unit cells with different lengths and widths and rotation angles in our simulation for this metalens, whose top view is shown in Figure 5a. We take polarization conversion efficiency into consideration except for the transmittance. Based on the database above, we select the unit cells with a polarization conversion higher than 85% (corresponding to $148.2{}^{\circ}<\left|\phi_{y}-\phi_{x}\right|<201.8{}^{\circ}$) to form a sub-database. The best unit cells with good performance at their positions (*x_i_*,*y_i_*) can be optimized in this database by minimizing $\left|\phi_{L}\left(x_{i},y_{i}\right)+\phi_{R}\left(x_{i},y_{i}\right)-(\phi_{x}^{C}+\phi_{y}^{C})\right|$. $\phi_{L}\left(x_{i},y_{i}\right)$ and $\phi_{R}\left(x_{i},y_{i}\right)$ are the required phases at (*x_i_*,*y_i_*) determined by Equation (2a,b).

The transmitted RCP and LCP light are focused at the positions of (−5 um, 0 um, 20 um) and (5 um, 0 um, 20 um) for the LCP and RCP incidence on the metalens, respectively. Their transmitted intensity distributions of the electric field are shown in Figure 5c,d, respectively. The FWHM of the focal spots are 700 nm for both LCP and RCP incidence as shown in Figure 5b. We also calculated the focusing efficiency and diffraction efficiency. The focusing efficiencies are 42.45% and 42.46% for LCP and RCP incidence, respectively. The diffraction efficiency can reach 63.40% for LCP and RCP light. A beam of x-linearly polarized light can be viewed as a superposition of LCP and RCP incidence light with an equal amplitude of $1/\sqrt{2}$. Hence, when a beam of x-linearly polarized light on the metalens, two focal spots will appear at the preset positions and the intensity distribution is shown in Figure 5e.

This work demonstrated that one can independently control the phases for two mutually orthogonal polarizations, x- and y-linearly polarized light, and LCP and RCP incidence light. The two designed metalenses can simultaneously form images for two orthogonal polarization basis sets.

## 4. Conclusions

In this study, we proposed an orthogonal linear polarization multiplexing metalens and an orthogonal circular polarization multiplexing metalens based on the independent phase control for two mutually orthogonal polarizations. These metalenses consist of 51 × 51 unit cells and can cover the entire phase range of 0~2π for orthogonal linear and circular polarizations, respectively. The linear polarization multiplexing metalens can independently focus x- and y-linearly polarized incident lights to arbitrary special positions with a focusing efficiency of 53.81% for x-linearly polarized light and 51.59% for y-linearly polarized light. A circular polarization multiplexing metalens with two preset focal points has also been designed and the focusing efficiency can reach 42.45% and 42.46% for LCP and RCP light. The two metalenses represent a unique platform for polarization optics and pave the way in polarization imaging technology.

## Data Availability

Data are contained within the article. The data presented in this study are available in Section 2 (Design and Method) and Section 3 (Simulation and Discussion).

## Supplementary Materials

The following supporting information can be downloaded at: https://www.mdpi.com/article/10.3390/nano12091500/s1, Table S1: The phase *ϕx* with different length (L) and width (W); Table S2: The transmittance T*x* with different length (L) and width (W).
