# Supplementary material for: High-Efficiency Polarization Multiplexing Metalenses
Source: Nanomaterials (Basel). 2022 Apr 28;12(9):1500. doi: 10.3390/nano12091500 (PMC9103879; doi:10.3390/nano12091500)
Supplement: Supplementary file 1 [file nanomaterials-12-01500-s001.zip › nanomaterials-1687650-supplementary.pdf]

nanomaterials

MDPI

Supplementary Materials

High-Efficiency Polarization Multiplexing Metalenses

Xueping Sun [1], Rui Ma [2], Xinxin Pu [1], Shaobo Ge [1], Jin Cheng [1], Xiangyang Li [3], Quan Wang [4], Shun Zhou [1,*] and Weiguo Liu [1,*]

1 Shanxi Province Key Laboratory of Thin Films Technology and Optical Test, School of Optoelectronic Engineering, Xi'an Technological University, Xi'an 710032, China; sunxueping@xatu.edu.cn (X.S.); puxinxin@st.xatu.edu.cn (X.P.); geshaobo@xatu.edu.cn (S.G.); chengjin36@xatu.edu.cn (J.C.)
2 School of Microelectronics, Xidian University, Xi'an 710071, China; marui@xatu.edu.cn
3 National Key Laboratory of Science and Technology on Space Microwave, China Academy of Space Technology (Xi'an), No.504 Dong chang'an Street, Xi'an 710100, China; lixiangyang@sina.com
4 Department of Biomedical Engineering, University of Strathclyde, Glasgow G11XQ, Scotland, UK; quan.wang.100@strath.ac.uk
* Correspondence: zhoushun@xatu.edu.cn (S.Z.); wgliu@163.com (W.L.)

Table S1. The phase ϕx with different length (L) and width (W).

L(μm) / W(μm)	0.07	0.08	0.09	0.1	0.11	0.12	0.13	0.14	0.15	0.16	0.17	0.18	0.19	0.2	0.21	0.22	0.23	0.24	0.25	0.26	0.27	0.28	0.29	0.3	0.31	0.32	0.33	0.34	0.35	0.36	0.37
0.07	3.01	3.10	3.18	3.27	3.36	3.48	3.59	3.69	3.82	3.97	4.11	4.23	4.40	4.55	4.72	4.90	5.07	5.28	5.38	5.33	1.06	0.33	0.37	0.46	0.56	0.67	0.75	0.83	0.93	1.03	1.14
0.08	3.05	3.16	3.23	3.33	3.45	3.58	3.71	3.83	3.98	4.16	4.33	4.48	4.70	4.92	5.13	5.38	5.51	5.21	0.52	0.45	0.59	0.71	0.82	0.95	1.05	1.18	1.29	4.56	5.76	1.07	1.43
0.09	3.10	3.21	3.30	3.42	3.55	3.69	3.84	3.98	4.15	4.37	4.59	4.80	5.09	5.38	5.62	5.60	0.54	0.59	0.71	0.86	1.01	1.14	1.26	4.56	4.97	1.29	1.52	1.66	1.77	1.89	1.99
0.1	3.14	3.26	3.36	3.49	3.63	3.79	3.96	4.12	4.33	4.59	4.88	5.15	5.55	5.81	4.92	0.61	0.74	0.93	1.08	1.22	4.49	4.45	2.01	1.92	2.03	2.16	2.30	2.43	2.61	2.77	2.97
0.11	3.17	3.31	3.42	3.56	3.71	3.89	4.08	4.27	4.52	4.86	5.23	5.59	6.00	1.08	0.68	0.88	1.05	1.23	4.41	3.97	2.49	2.40	2.53	2.70	2.85	3.04	3.19	3.32	3.49	3.62	3.82
0.12	3.21	3.35	3.46	3.61	3.78	3.98	4.20	4.40	4.70	5.12	5.56	5.93	6.12	0.71	0.88	1.09	4.41	4.36	3.33	2.62	2.69	2.86	3.02	3.21	3.36	3.52	3.64	3.75	3.90	4.04	4.41
0.13	3.24	3.40	3.52	3.68	3.86	4.08	4.31	4.56	4.91	5.42	5.92	6.21	0.79	0.89	1.07	1.22	4.19	3.13	2.85	2.94	3.13	3.30	3.44	3.60	3.72	3.86	3.95	4.05	4.21	4.47	3.37
0.14	3.28	3.44	3.57	3.74	3.94	4.17	4.44	4.73	5.16	5.75	6.25	4.76	0.86	1.07	1.19	4.19	3.25	3.03	3.13	3.28	3.47	3.61	3.72	3.86	3.96	4.07	4.16	4.27	4.51	2.76	4.10
0.15	3.31	3.48	3.62	3.79	4.00	4.26	4.56	4.89	5.39	6.03	0.19	0.78	1.01	1.19	4.28	3.46	3.09	3.21	3.36	3.51	3.69	3.81	3.91	4.03	4.12	4.24	4.33	4.48	2.02	4.00	4.30
0.16	3.34	3.52	3.66	3.85	4.07	4.36	4.69	5.06	5.62	6.26	0.30	0.87	1.13	4.36	3.93	3.17	3.18	3.38	3.53	3.67	3.83	3.95	4.04	4.16	4.25	4.38	4.49	1.62	3.77	4.28	4.46
0.17	3.37	3.56	3.71	3.90	4.14	4.45	4.82	5.25	5.85	0.19	1.17	0.99	1.23	4.18	3.40	3.20	3.33	3.54	3.68	3.81	3.96	4.07	4.16	4.27	4.37	4.52	1.53	2.31	4.26	4.48	4.59
0.18	3.40	3.59	3.75	3.96	4.21	4.54	4.96	5.44	6.07	0.35	0.90	1.10	4.41	3.74	3.23	3.30	3.46	3.66	3.80	3.92	4.06	4.16	4.25	4.37	4.47	1.49	1.71	3.92	4.53	4.68	4.77
0.19	3.42	3.63	3.79	4.00	4.27	4.63	5.09	5.61	6.24	0.46	0.98	1.18	4.33	3.38	3.23	3.40	3.57	3.77	3.89	4.01	4.15	4.25	4.34	4.47	4.55	1.66	2.31	4.46	4.70	4.84	4.94
0.2	3.45	3.66	3.84	4.06	4.34	4.74	5.24	5.80	0.13	0.37	1.08	1.24	4.03	3.25	3.30	3.51	3.68	3.86	3.99	4.10	4.24	4.34	4.43	4.56	1.54	1.81	4.31	4.77	4.95	5.08	5.19
0.21	3.48	3.70	3.88	4.11	4.40	4.83	5.37	5.95	0.25	1.18	1.15	1.25	3.73	3.24	3.36	3.58	3.75	3.93	4.05	4.16	4.30	4.41	4.51	4.63	1.63	2.34	4.70	4.91	5.06	5.19	5.30
0.22	3.50	3.73	3.91	4.15	4.46	4.92	5.51	6.09	0.36	1.00	1.23	4.36	3.44	3.27	3.43	3.66	3.82	4.00	4.12	4.24	4.38	4.50	4.60	1.63	1.83	4.20	4.77	4.95	5.09	5.21	5.24
0.23	3.54	3.77	3.96	4.21	4.54	5.04	5.66	6.23	0.46	1.04	1.30	4.21	3.29	3.33	3.51	3.73	3.89	4.07	4.19	4.31	4.46	4.59	4.72	1.82	2.38	4.16	4.47	4.56	4.45	4.14	0.54
0.24	3.56	3.80	3.99	4.26	4.60	5.14	5.78	0.06	0.52	1.11	1.35	3.97	3.26	3.39	3.58	3.80	3.95	4.13	4.25	4.38	4.56	4.73	4.98	5.59	6.05	6.07	5.94	5.85	5.78	5.77	5.76
0.25	3.58	3.82	4.02	4.29	4.66	5.23	5.89	0.15	0.56	1.16	1.36	3.68	3.26	3.44	3.62	3.84	4.00	4.18	4.32	4.48	4.87	0.51	0.75	5.70	5.26	5.13	5.02	4.97	4.77	1.46	1.27
0.26	3.62	3.86	4.07	4.35	4.75	5.35	6.02	0.26	0.48	1.23	1.36	3.43	3.28	3.49	3.67	3.89	4.06	4.37	4.30	4.43	4.60	1.67	4.07	4.39	1.15	1.01	0.90	0.79	0.73	0.81	0.84
0.27	3.64	3.89	4.10	4.40	4.81	5.45	6.12	0.33	1.83	1.30	4.44	3.26	3.29	3.51	3.67	4.04	4.09	4.26	4.34	3.44	4.72	4.68	1.46	1.15	0.73	0.46	0.38	0.36	0.39	0.37	0.94
0.28	3.66	3.92	4.14	4.45	4.89	5.56	6.23	0.42	1.13	1.36	4.22	3.14	3.26	3.10	3.85	4.00	4.05	4.42	4.53	4.66	4.83	4.90	1.83	1.91	2.00	4.63	4.55	4.43	4.30	4.21	4.28
0.29	3.69	3.96	4.18	4.50	4.96	5.65	0.02	0.47	1.11	1.40	3.75	2.93	4.48	3.74	3.82	4.07	4.21	4.41	4.55	4.71	4.93	5.18	2.43	3.09	3.47	0.55	0.63	0.69	0.76	0.82	4.91
0.3	3.71	3.98	4.21	4.54	5.03	5.74	0.10	0.53	1.14	1.44	2.64	4.73	3.64	3.83	3.90	4.09	4.24	4.44	4.60	4.78	5.11	5.56	6.05	0.15	0.32	0.46	0.54	0.61	0.70	0.82	0.12
0.31	3.74	4.02	4.26	4.61	5.12	5.85	0.19	0.59	1.19	1.44	1.75	3.76	3.67	3.79	3.93	4.12	4.27	4.43	4.57	4.85	5.45	5.96	6.28	0.20	0.32	0.43	0.49	0.56	0.65	0.85	6.25
0.32	3.76	4.05	4.29	4.66	5.20	5.94	0.27	0.63	1.25	1.65	4.39	0.32	3.68	3.83	3.96	4.09	4.06	5.68	5.40	4.33	6.24	6.25	0.10	0.21	0.28	0.34	0.36	0.37	0.37	0.93	2.45
0.33	3.77	4.07	4.32	4.70	5.27	6.02	0.33	0.65	1.31	1.58	4.62	3.53	3.74	3.82	3.99	4.90	4.62	4.90	5.06	4.71	0.81	0.37	0.19	0.17	6.27	5.63	1.73	1.35	1.13	0.96	1.18
0.34	3.81	4.11	4.37	4.77	5.37	6.12	0.40	0.61	1.68	1.57	3.66	3.60	3.80	3.77	4.23	4.43	4.64	4.84	4.99	4.83	1.48	1.62	1.46	0.77	0.74	0.86	0.90	0.89	0.97	4.21	0.85
0.35	3.82	4.14	4.40	4.82	5.45	6.21	0.46	0.31	1.39	1.89	3.67	3.73	4.67	3.98	4.21	4.43	4.63	4.82	4.80	1.62	1.83	5.65	0.04	0.43	0.65	0.76	0.83	0.90	0.96	4.88	1.10
0.36	3.85	4.16	4.45	4.89	5.54	0.04	0.55	1.96	1.51	4.35	3.64	3.87	3.83	4.08	4.26	4.46	4.62	1.40	1.39	1.56	5.48	2.21	0.12	0.51	0.63	0.46	0.83	0.91	1.01	4.34	0.86
0.37	3.87	4.19	4.49	4.95	5.63	0.11	0.60	1.39	1.64	4.22	3.86	3.29	3.87	4.04	4.31	4.45	1.27	1.13	1.23	1.66	1.90	6.03	0.19	0.55	0.62	0.80	0.92	1.00	1.04	4.25	1.89

Table S2. The transmittance Tx with different length (L) and width (W).

L(μm) W(μm)	0.07	0.08	0.09	0.1	0.11	0.12	0.13	0.14	0.15	0.16	0.17	0.18	0.19	0.2	0.21	0.22	0.23	0.24	0.25	0.26	0.27	0.28	0.29	0.3	0.31	0.32	0.33	0.34	0.35	0.36	0.37
0.07	0.97	0.97	0.97	0.98	0.98	0.97	0.97	0.97	0.97	0.97	0.97	0.97	0.98	0.98	0.99	0.99	0.98	0.97	0.94	0.77	0.83	0.99	0.98	0.97	0.96	0.95	0.95	0.95	0.96	0.98	0.99
0.08	0.97	0.97	0.98	0.98	0.97	0.97	0.97	0.97	0.97	0.97	0.97	0.98	0.98	0.99	0.98	0.97	0.91	0.26	0.97	0.98	0.97	0.96	0.96	0.96	0.96	0.94	0.82	0.52	0.98	0.99	1.00
0.09	0.97	0.97	0.97	0.97	0.97	0.97	0.97	0.97	0.97	0.97	0.98	0.98	0.98	0.98	0.94	0.67	0.96	0.97	0.97	0.96	0.95	0.87	0.26	0.78	0.96	0.99	1.00	0.99	0.96	0.93	0.89
0.1	0.97	0.97	0.97	0.97	0.97	0.97	0.97	0.97	0.97	0.98	0.98	0.99	0.97	0.90	0.00	0.97	0.96	0.95	0.90	0.32	0.74	0.96	0.99	1.00	0.99	0.96	0.94	0.91	0.86	0.83	0.78
0.11	0.97	0.97	0.97	0.97	0.97	0.97	0.97	0.97	0.97	0.98	0.98	0.97	0.91	0.52	0.95	0.94	0.88	0.20	0.74	0.95	0.99	0.99	0.99	0.96	0.94	0.90	0.88	0.85	0.82	0.81	0.80
0.12	0.97	0.97	0.97	0.97	0.97	0.97	0.97	0.97	0.98	0.98	0.98	0.95	0.64	0.94	0.94	0.84	0.00	0.83	0.96	0.99	0.98	0.97	0.95	0.92	0.90	0.87	0.86	0.85	0.84	0.85	0.85
0.13	0.97	0.97	0.97	0.97	0.97	0.97	0.97	0.97	0.98	0.98	0.96	0.86	0.81	0.92	0.81	0.00	0.84	0.96	0.98	0.98	0.95	0.93	0.92	0.90	0.89	0.88	0.89	0.90	0.90	0.45	0.92
0.14	0.97	0.97	0.97	0.97	0.97	0.97	0.97	0.98	0.98	0.98	0.94	0.00	0.90	0.79	0.07	0.74	0.94	0.97	0.97	0.95	0.93	0.91	0.90	0.89	0.89	0.89	0.88	0.80	0.01	0.92	1.00
0.15	0.97	0.97	0.97	0.97	0.97	0.97	0.97	0.98	0.99	0.97	0.89	0.83	0.84	0.26	0.47	0.90	0.96	0.96	0.94	0.92	0.89	0.87	0.86	0.84	0.80	0.73	0.54	0.03	0.82	1.00	0.98
0.16	0.97	0.97	0.97	0.97	0.97	0.97	0.97	0.98	0.98	0.96	0.69	0.87	0.70	0.11	0.77	0.94	0.95	0.93	0.90	0.87	0.84	0.80	0.77	0.71	0.59	0.35	0.03	0.48	0.97	0.98	0.92
0.17	0.97	0.97	0.97	0.97	0.97	0.97	0.98	0.99	0.98	0.95	0.19	0.83	0.33	0.57	0.87	0.94	0.93	0.89	0.85	0.80	0.75	0.67	0.60	0.47	0.28	0.02	0.18	0.84	0.97	0.92	0.85
0.18	0.97	0.97	0.97	0.97	0.97	0.97	0.98	0.99	0.98	0.92	0.74	0.70	0.00	0.76	0.89	0.91	0.88	0.82	0.76	0.69	0.60	0.49	0.38	0.24	0.07	0.05	0.54	0.94	0.92	0.85	0.79
0.19	0.97	0.97	0.97	0.97	0.97	0.97	0.98	0.99	0.97	0.87	0.78	0.51	0.21	0.84	0.90	0.88	0.83	0.76	0.67	0.57	0.48	0.35	0.24	0.10	0.00	0.32	0.79	0.94	0.87	0.81	0.74
0.2	0.97	0.97	0.97	0.97	0.97	0.97	0.98	0.99	0.96	0.27	0.73	0.16	0.53	0.86	0.87	0.82	0.73	0.64	0.52	0.42	0.33	0.22	0.12	0.03	0.04	0.55	0.87	0.91	0.83	0.77	0.73
0.21	0.97	0.97	0.97	0.97	0.97	0.98	0.99	0.98	0.95	0.17	0.67	0.01	0.65	0.87	0.85	0.77	0.66	0.56	0.44	0.34	0.26	0.15	0.07	0.00	0.15	0.72	0.89	0.88	0.80	0.76	0.73
0.22	0.97	0.97	0.97	0.97	0.97	0.98	0.99	0.98	0.95	0.58	0.57	0.08	0.74	0.85	0.80	0.70	0.57	0.47	0.36	0.27	0.20	0.11	0.04	0.01	0.29	0.78	0.86	0.84	0.79	0.77	0.75
0.23	0.97	0.97	0.97	0.97	0.97	0.98	0.99	0.98	0.94	0.69	0.39	0.30	0.78	0.82	0.73	0.60	0.47	0.38	0.29	0.21	0.17	0.08	0.02	0.04	0.39	0.73	0.78	0.77	0.65	0.06	0.25
0.24	0.97	0.97	0.97	0.97	0.97	0.98	0.99	0.98	0.91	0.69	0.24	0.47	0.80	0.79	0.68	0.55	0.42	0.35	0.28	0.22	0.21	0.15	0.12	0.34	0.92	0.82	0.75	0.68	0.61	0.56	0.49
0.25	0.97	0.97	0.97	0.97	0.97	0.98	0.99	0.98	0.80	0.66	0.09	0.58	0.79	0.74	0.61	0.49	0.38	0.36	0.38	0.62	0.18	0.04	0.11	0.48	0.79	0.73	0.62	0.44	0.03	0.53	0.96
0.26	0.97	0.97	0.96	0.96	0.97	0.98	0.99	0.97	0.41	0.61	0.00	0.67	0.78	0.69	0.55	0.47	0.58	0.01	0.02	0.03	0.01	0.00	0.17	0.04	0.39	0.89	0.95	0.94	0.93	0.92	0.90
0.27	0.97	0.97	0.97	0.96	0.97	0.99	0.99	0.97	0.04	0.55	0.03	0.73	0.77	0.66	0.66	0.06	0.09	0.09	0.06	0.01	0.05	0.00	0.06	0.42	0.74	0.86	0.88	0.89	0.89	0.89	0.67
0.28	0.97	0.97	0.96	0.97	0.97	0.99	0.99	0.96	0.43	0.46	0.18	0.76	0.76	0.22	0.18	0.12	0.04	0.36	0.15	0.09	0.05	0.00	0.07	0.39	0.64	0.75	0.77	0.76	0.67	0.39	0.56
0.29	0.97	0.97	0.96	0.97	0.98	0.99	0.99	0.96	0.56	0.37	0.38	0.84	0.20	0.28	0.08	0.81	0.28	0.19	0.15	0.12	0.10	0.03	0.00	0.05	0.03	0.06	0.27	0.46	0.62	0.70	0.86
0.3	0.97	0.97	0.96	0.97	0.98	0.99	0.99	0.96	0.62	0.24	0.74	0.08	0.39	0.01	0.62	0.31	0.21	0.22	0.25	0.38	0.93	0.86	0.92	0.98	0.91	0.84	0.83	0.82	0.84	0.85	0.86
0.31	0.97	0.97	0.96	0.97	0.98	0.99	0.98	0.96	0.63	0.05	0.15	0.51	0.58	0.59	0.35	0.25	0.23	0.67	0.26	0.06	0.08	0.27	0.64	0.87	0.90	0.88	0.87	0.87	0.87	0.87	0.87
0.32	0.97	0.97	0.96	0.97	0.98	0.99	0.98	0.95	0.61	0.96	0.22	0.03	0.66	0.44	0.29	0.30	0.04	0.02	0.01	0.00	0.03	0.23	0.60	0.83	0.87	0.87	0.86	0.85	0.81	0.63	0.04
0.33	0.97	0.97	0.96	0.97	0.98	0.99	0.98	0.93	0.57	0.27	0.31	0.73	0.55	0.35	0.28	0.09	0.07	0.06	0.03	0.00	0.03	0.23	0.57	0.80	0.84	0.65	0.54	0.82	0.83	0.22	0.97
0.34	0.97	0.96	0.96	0.97	0.98	0.99	0.98	0.80	0.26	0.06	0.52	0.65	0.47	0.29	0.18	0.13	0.09	0.08	0.04	0.00	0.06	0.36	0.65	0.78	0.86	0.86	0.88	0.85	0.81	0.40	0.96
0.35	0.97	0.96	0.96	0.97	0.98	0.99	0.97	0.24	0.62	0.02	0.56	0.60	0.46	0.24	0.17	0.14	0.10	0.08	0.02	0.09	0.22	0.16	0.57	0.80	0.80	0.86	0.83	0.81	0.63	0.97	0.99
0.36	0.97	0.96	0.96	0.97	0.99	1.00	0.97	0.14	0.51	0.03	0.58	0.55	0.32	0.20	0.15	0.13	0.09	0.10	0.30	0.12	0.00	0.04	0.51	0.73	0.79	0.57	0.81	0.79	0.61	0.96	0.95
0.37	0.97	0.96	0.96	0.97	0.99	0.99	0.97	0.41	0.42	0.09	0.59	0.35	0.27	0.17	0.13	0.11	0.35	0.23	0.17	0.06	0.01	0.16	0.52	0.68	0.78	0.78	0.77	0.72	0.36	0.99	0.89
